# The Predictive Role of Lactate in the Emergency Department in Patients with Severe Dyspnea

**DOI:** 10.1155/2024/6624423

**Published:** 2024-02-29

**Authors:** Maciej Niczewski, Szymon Gawęda, Paulina Kluszczyk, Mikołaj Rycerski, Daria Syguła, Anna Danel, Szymon Szmigiel, Konrad Mendrala, Aleksandra Oraczewska, Czarosław Kijonka, Monika Nowicka, Michał Wita, Tomasz Cyzowski, Grzegorz Brożek, Maciej Dyrbuś, Szymon Skoczyński

**Affiliations:** ^1^Department of Internal Medicine and Metabolic Diseases, Faculty of Health Sciences in Katowice, Medical University of Silesia, Katowice, Poland; ^2^Student Scientific Society, Department of Lung Diseases and Tuberculosis, Faculty of Medical Sciences in Zabrze, Medical University of Silesia, Katowice, Poland; ^3^Department of Lung Diseases and Tuberculosis, Faculty of Medical Sciences in Zabrze, Medical University of Silesia, Katowice, Poland; ^4^1st Department of Lung Diseases and Tuberculosis, Provincial Specialist Hospital in Czerwona Góra, Chęciny, Poland; ^5^Department of Anaesthesiology and Intensive Care, Medical University of Silesia, Katowice, Poland; ^6^Emergency Department, Upper Silesian Medical Center, Faculty of Health Sciences in Katowice, Medical University of Silesia, Katowice, Poland; ^7^First Chair and Department of Cardiology, Faculty of Medical Sciences in Katowice, Medical University of Silesia, Katowice, Poland; ^8^Department of Epidemiology, Faculty of Medical Sciences in Katowice, Medical University of Silesia, Katowice, Poland; ^9^3rd Department of Cardiology, Faculty of Medical Sciences in Zabrze, Medical University of Silesia, Katowice, Poland

## Abstract

**Objective:**

An accurate identification of patients at the need for prioritized diagnostics and care are crucial in the emergency department (ED). Blood gas (BG) analysis is a widely available laboratory test, which allows to measure vital parameters, including markers of ventilation and perfusion. The aim of our analysis was to assess whether blood gas parameters in patients with dyspnea at an increased risk of respiratory failure admitted to the ED can predict short-term outcomes.

**Methods:**

The study group eventually consisted of 108 patients, with available BG analysis. The clinical and laboratory parameters were retrospectively evaluated, and three groups were distinguished—arterial blood gas (ABG), venous blood gas (VBG), and mixed blood gas. The primary endpoint was short-term, all-cause mortality during the follow-up of median (quartile 1–quartile 3) 2 (1–4) months. The independent risk factors for mortality that could be obtained from blood gas sampling were evaluated.

**Results:**

The short-term mortality was 35.2% (38/108). Patients who died were more frequently initially assigned to the red triage risk group, more burdened with comorbidities, and the median SpO_2_ on admission was significantly lower than in patients who survived the follow-up period. In the multivariable analysis, lactate was the strongest independent predictor of death, with 1 mmol/L increasing all-cause mortality by 58% in ABG (95% CI: 1.01–2.47), by 80% in VBG (95% CI: 1.13–2.88), and by 68% in the mixed blood gas analysis (95% CI: 1.22–2.31), what remained significant in VBG and mixed group after correction for base excess. In each group, pH, pO_2_, and pCO_2_ did not predict short-term mortality.

**Conclusions:**

In patients admitted to the ED due to dyspnea, at risk of respiratory failure, lactate levels in arterial, venous, and mixed blood samples are independent predictors of short-term mortality.

## 1. Introduction

Blood gas sample analysis is often used in the intensive care unit (ICU), emergency department (ED), pulmonary department, and others to assess the respiratory capacity and acid-base balance. Parameters such as pH, pO_2_, pCO_2_, and lactate can be measured in arterial, venous, or capillary whole blood samples [1]. Elevated blood lactate levels reflect circulatory shock or ongoing tissue hypoxia in patients [2]. Impaired perfusion can lead to multiple organ failure and death; that is why it is crucial to reliably assess whether the patient is hemodynamically stable. Capillary refill and perfusion or diuresis are also evaluated; however, monitoring of lactate concentration may provide more specific results. Furthermore, the appropriate treatment has a quick impact on the lactate level which makes the measurement even more valuable as a tissue perfusion biomarker [3]. It is known that regardless of its source, an elevated lactate level is associated with worse outcomes, and lactate clearance was found to be a predictor of lower mortality in critically ill patients [4].

Dyspnea is one of the most significant symptoms reported by the patients on admission to the ED and can be caused by increased metabolic demand, decreased chest compliance, or increased dead-space volume, occurring in multiple disorders, including pulmonary, cardiovascular, or neurological diseases [5]. It has been reported that patients with dyspnea have a higher risk of a readmission to the ED in comparison to patients without such symptoms, and although a nonspecific symptom, dyspnea has been proven as an independent risk factor for all-cause mortality [6, 7]. It is thus demanding for clinicians to evaluate the dyspneic patient correctly and provide appropriate treatment.

An efficient workflow and appropriate identification of patients at the highest need for prioritized diagnostics and care are crucial in the setting of ED. Triage is the first step to identify subjects in a life-threatening condition and those with lower priority for rapid diagnostics. Extension of the standard triage system by an addition of quick laboratory tests like blood gas (BG) samples may increase the accuracy of medical risk stratification. The aim of this study was to identify parameters of blood gas analysis, which would have a predictive value on prognosis in patients at risk of respiratory failure admitted to the ED.

## 2. Materials and Methods

### 2.1. Study Design

We concluded a retrospective study enrolling patients with dyspnea and a risk of respiratory failure, admitted to the ED of a multispeciality, academic, 700-bedded hospital. The approval of a bioethics committee was not required based on the decision made by the head of the Bioethical Board of the Medical University of Silesia (PCN/CBN/0052/KB/154/22).

### 2.2. Study Setting and Population

We obtained data from medical files of patients admitted to the ED during 3 consecutive months, between 01 September 2022 and 30 November 2022. We included patients with infectious pulmonary diseases, dyspnea, exacerbation of chronic heart failure, and neurological disorders. Exclusion criteria were acute coronary syndrome (ACS), arrhythmias, and posttraumatic disorders. We also excluded patients who had been intubated by an emergency medical team and mechanically ventilated prior to the admission to the ED. Of the entire analyzed population, the study group consisted solely of patients with BG results available for analysis. The decision whether a patient should or should not have undergone the BG test was at the discretion of the treating ED physician based on initial assessment and physical examination. Three groups of data were distinguished—arterial blood gas (ABG), venous blood gas (VBG), and mixed. The latter group consists of patients with ABG and VBG and those patients with unknown FiO_2_; therefore, none of them could be assigned to ABG or VBG groups. The primary endpoint was short-term mortality, with a median (quartile 1–quartile 3) follow-up period of 2 (1–4) months. The occurrence of the primary endpoint was verified based on data from the electronic databases of the National Health Fund (*Narodowy Fundusz Zdrowia*—*NFZ*), the primary healthcare provider in Poland.

### 2.3. Triage

All patients admitted to ED were triaged using the “TOPSOR” triage system [8]. TOPSOR is based on the Emergency Severity Index algorithm. It segregates patients into 5 colour-coded levels according to urgency of emergency and resources expected to be required to diagnose and treat the patient's condition. Stratification was performed by a nurse, a paramedic, or a doctor based on medical history and clinical status evaluation. If needed, it was supplemented by measurement of capillary blood glucose, body temperature, and an electrocardiogram. Of 5 potential risk categories, patients with shortness of breath would be usually assigned into yellow, orange, or red categories, meaning a necessity of evaluation by a doctor maximally within 60 minutes from admission.

### 2.4. Analysis

StatsDirect 3.1 (StatsDirect Ltd. Wirral, UK) was used for statistical analysis. The distribution of variables was based on the Shapiro–Wilk test and QQ plot analysis.

For logistic regression, potential risk factors were chosen based on parameters typically available in blood gas analyzers—pO_2_, pCO_2_, HCO_3_^−^, sO_2_, BE, Lac, and P/F. Spearman correlation coefficients were determined, and only variables with correlations <0.7 were included in the analysis. Univariable logistic regression was performed, based on which the independent variables with the highest OR/value of the Wald test were selected at the level of significance 0.25. We conduct a purposeful selection of variables as per Bursac et al. [9]. In the binominal regression model, significance of variables was determined at the 0.1 alpha level, while confounding was defined as a change in the remaining parameter of more than 15%. When covariates were nonsignificant and not cofounders, they were eliminated from the model. Model evaluation was based on the Hosmer–Lemeshow test and McFadden pseudo *R* Square. The comparison of the models was based on the AUC.

In descriptive statistics, quantitative variables are presented as the median and interquartile range (IQR, interquartile range). Qualitative variables are presented as absolute values and percentages. Differences between groups were assessed using the Mann–Whitney *U* test. For qualitative variables, contingency tables and the chi-square or Fisher's exact test were used. We assumed two-tailed *p* < 0.05 to be statistically significant.

## 3. Results

### 3.1. Characteristics of the Study Participants

**Figure 1:**
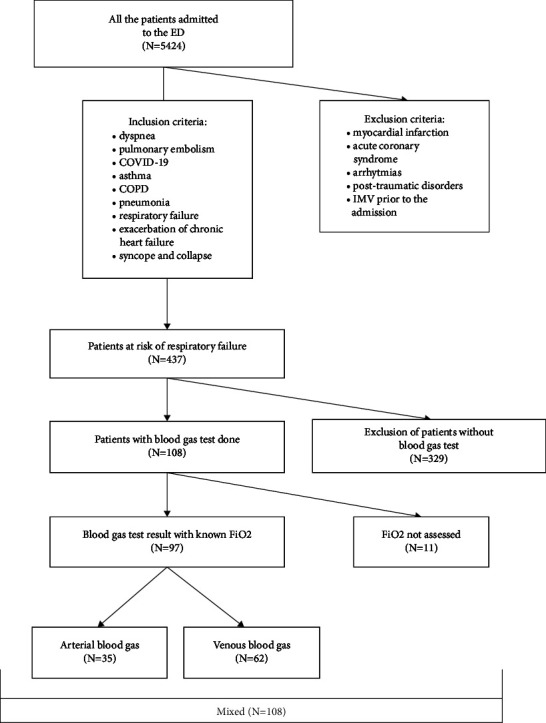
Flowchart for the selection of patients.

Among all patients admitted to the ED during 3 months (total *n* = 5424), there were 437 patients meeting the inclusion criteria. From that group, we have identified those, in whom the blood gas test was ordered, who thus constituted the study group of 108 patients (Figure 1). The study population was divided into three groups based on the type of BG analysis (ABG, VBG, and mixed). During the median (quartile 1–quartile 3) follow-up of 2 (1–4) months, the all-cause mortality rate was 35.2% (38/108).

The median age of patients who survived was 71. The majority (70%) of surviving patients received yellow colour after triage. Of patients who survived, 41% came to the ED by themselves, and in 51%, the primary reason for presentation to the ED was dyspnea. 66% of patients who eventually survived were transferred to a different ward, and their median (Q1–Q3) time spent at the ED was 5 (3.5–8.5) hours. Among comorbidities, 59% had previously diagnosed hypertension (HT), 69% were not on oxygen support during BG analysis, and 55% were not on oxygen support during their stay at the ED.

In the group of patients who died, their age median was 79.5 (69–87), most of whom were yellow in triage (39%) and were transported to the ED by emergency medical services (74%), in 37%, their reason of admission was heart failure, while 58% had previously diagnosed HT, and eventually, 61% were transferred to a different ward. The median (Q1–Q3) time spent at the ED was 6.75 (4–12) hours, and 24% of those patients were not on oxygen support during BG analysis, while the other 68% were on oxygen therapy during their stay at the ED (Table 1). Among all patients, 8 of 108 patients were transferred to the ICU (7.4% of the study group). 6 of them died, 4 patients in the ICU and 2 after discharge.

### 3.2. Main Results

The summary of ABG results is presented in Tables 2 and 3. The summarized VBG results are presented in Tables 4 and 5. Of the 108 patients, three groups of data were distinguished—arterial blood gas (*n* = 35), venous blood gas (*n* = 62), and mixed, which included both ABG and VBG as well as those patients in whom FiO_2_ could not be assessed (*n* = 11). For final analysis, three parameters were included in the regression model—Lac, HCO_3_^−^, or BE. In each group model, lactate was the strongest risk factor for death: the odds ratios (OR) for higher risk of death with increasing lactates were 1.58 (1.01–2.47) for ABG, 1.8 (1.13–2.88) for VBG, and 1.68 (1.22–2.31) for ABG/VBG. The ROC analysis of results from the ABG/VBG group is shown in Table 6. The AUC in the ROC curves for lactates was 0.81 in the ABG group, 0.68 in the VBG group, and 0.75 in the ABG/AVG group.

**Figure 2:**
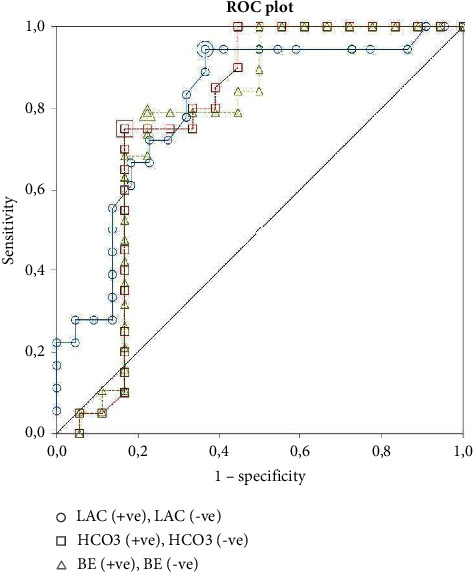
ROC plot for ABG results. LAC: cutoff 1.5 mmol/L (sensitivity = 0.94 (0.73–0.99), specificity = 0.64 (0.41–0.83)), AUC 0.81; HCO_3_^−^: cutoff 24.7 mmol/L (sensitivity = 0.75 (0.51–0.91), specificity = 0.83 (0.59–0.96)), AUC 0.7; BE: cutoff −0.9 (sensitivity = 0.79 (0.54–0.94), specificity = 0.78 (0.52–0.96)), AUC 0.77.

**Figure 3:**
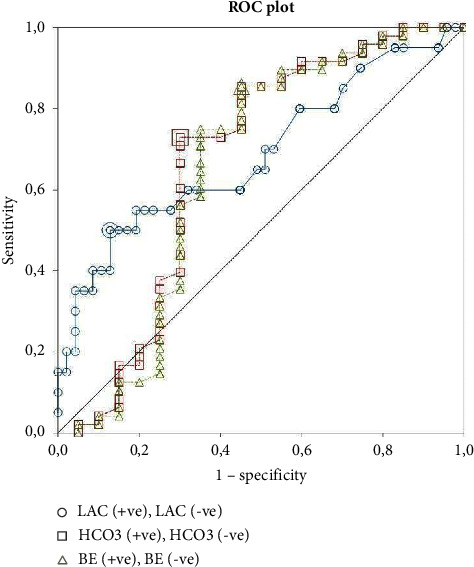
ROC plot for VBG results. LAC: cutoff 3.3 mmol/L, sensitivity = 0.47 (0.23 to 0.72), specificity = 0.91 (0.78 to 0.97), AUC 0.68; HCO_3_^−^: cutoff 23.4 mmol/L, sensitivity = 0.71 (0.44 to 0.90), specificity = 0.76 (0.60 to 0.87), AUC 0.67; BE: cutoff −0.4 mmol/L, sensitivity = 0.65 (0.38 to 0.86), specificity = 0.78 (0.63 to 0.89), AUC 0.65.

**Figure 4:**
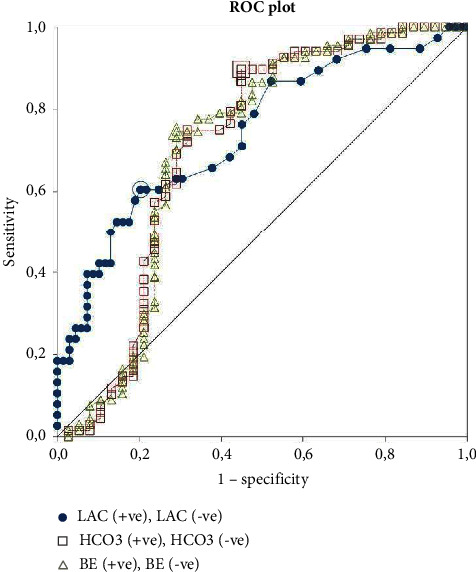
ROC plot for ABG/VBG results. LAC: cutoff 2.5 mmol/L (sensitivity = 0.61 (0.43–0.76), specificity = 0.80 (0.68–0.88)), AUC 0.74; HCO_3_^−^: cutoff 21.3 mmol/L (sensitivity = 0.90 (0.80–0.96), specificity = 0.55 (0.38–0.71)), AUC 0.71; BE: cutoff −0.3 mmol/L (sensitivity = 0.75 (0.63–0.84), specificity = 0.71 (0.54–0.85)), AUC 0.70.

When corrected for BE, VBG lactate OR was 1.38 (1.05–1.80), *p*=0.46, and ABG/VBG lactate corrected for BE was 1.41 (1.11–1.80), *p*=0.28. In the ABG group, the model containing lactate and BE or lactate and HCO_3_ had worse prediction than the individual variables—BE corrected lactate OR was 1.67 (0.86–3.25), *p*=0.097, McFadden *R*-square 0.31. A detailed analysis of the ABG regression parameters is shown in Table 7. ROC curves for each variable are presented in Figures 2–4.

## 4. Discussion

Triage is a procedure aiming to stratify the risk of patients coming to emergency in order to prioritize adequate medical response. Organization of the ED depends on its effectiveness and readiness for massive patient inflow. Since the introduction of triage, many systems have been designed with an effort to provide quick and precise patient evaluation; however, their limitations are widely known. In addition, adaptation of the triage system varies internationally and limits the capacity for review and comparison [10, 11].

Dyspnea is a common complaint among ED patients, accounting for approximately four million visits (3%) annually in the United States [12]. It is critical at the ED to evaluate if a patient is in a life-threatening condition. Among signs and symptoms, the severity of shortness of breath, duration of symptoms, comorbidities, abnormalities on auscultation, and symptoms of fluid overload should be assessed. Pulse oximetry is also commonly used as an easily accessible supplementary method to rapidly assess the severity of dyspnea [13].

It is discussed whether primary disease responsible for dyspnea corresponds with the risk of mortality, but on the other hand, there are also results that diseases like pneumonia, COPD, and respiratory failure have higher risk of mortality among acute state patients hospitalized on the internal medicine ward [7, 14]. In our research, type 2 diabetes, asthma, malignancy, and chronic kidney disease were more prevalent in patients who died. Furthermore, it was established that the multiparameter score which consisted of 80 biomarkers from the Olink CVD1 panel plays a superior role in predicting the short- and long-term mortality than the multimorbidity score, consisting of any out of 22 previously predefined diseases [15]. In another analysis, biomarkers such as NT-proBNP, hs-cTnT, hs-CRP, and cystatin C (Cys-C) were used to stratify the risk of mortality in patients with dyspnea [16]. In said research, the MARKED (Multi mARKer Emergency Dyspnea)-risk score consisting of comorbidities, present symptoms, blood pressure, and hs-CRP, hs-cTnT, and Cys-C had advantages over a single-risk factor score. However, the MARKED-risk score needs time for complete biochemical evaluation, which is extremely important in the ED. According to our study, dyspnea or symptoms of HF on admission, as well as systolic blood pressure lower than 110 mmHg, were significantly more prevalent among patients who died.

The blood gas lactate level has been defined in our analysis as a risk factor of mortality among patients with dyspnea. The blood gas analysis while awaiting for the results of laboratory tests shortens the time needed for the initial assessment of the patient's condition and enables faster treatment. Lactate monitoring is increasingly being performed in critically ill patients because of its prognostic significance and a possibility to perform it almost at the patient's bedside [17–20]. Studies have shown that ICU patients with higher lactate levels on admission are associated with increased mortality mainly in older patients [21]. Other studies report that an initial lactate level at or above 3.0 mEq/L, which should be aimed to reduce by at least 20% per 2 hours, significantly shortens the length of ICU stay, and reduces hospital mortality [21, 22]. The concept of lactate clearance as a predictor of mortality among critically ill patients has been introduced, but the opinions on its utility are divided [23, 24]. According to some research, a low prehospital lactate clearance in patients with septic shock may be associated with a higher mortality. There have not been many studies evaluating the prognostic value of hyperlactatemia in patients with dyspnea. In our study, blood lactates were the strongest independent predictor of death, increasing the risk of death by 58% in the arterial BG (95% CI: 1.01–2.47), by 80% in the venous BG (95% CI: 1.13–2.88), and by 68% in the mixed group (95% CI: 1.22–2.31), per 1 mmol/L, what remained significant in VBG and mixed group after correction.

The gold standard for measurement of oxygen saturation remains arterial blood gas analysis. In our research, we have used ABG and peripheral venous blood gas (PVBG) samples. Arterial blood sampling could cause delay in patient treatment due to blood collection difficulties. In order to shorten the time of diagnosis, many researchers wanted to assess the differences between the results of ABG, capillary blood gas (CBG), and VBG samples. There are some similarities between capillary blood taken from hyperemized earlobes (CBGE) or fingertips (CBGF) and peripheral venous blood used as a substitute for ABG. The pH and pCO_2_ levels are similar in ABG and CBGE/CBGF, but on the other side, the level of pO_2_ is often underestimated by CBG. There were also invented software tools for mathematical arterialization of capillary blood samples and venous blood samples for blood gas analysis resulting in increased diagnostic accuracy for pO_2_. Using the mathematical arterialization by v-TAC, the disparity of pO_2_ between ABG and CBG was 0.18 mmHG [25]. The difference between the measured arterial pO_2_ and the mathematical arterialization of venous blood gas pO_2_ is higher in patients with higher SpO_2_. Exclusion of patients with SpO_2_ > 97% resulted in a better correlation between those data [26]. Studies also revealed that the arterial lactate level and the peripheral venous lactate level are not perfectly identical, but they are strongly correlated with each other [27]. Furthermore, scientists have found not only the peripheral venous but also central venous pH, bicarbonate, base excess, and lactate values have almost 95% limit of agreement with arterial blood samples [28]. According to this state, we think all blood gas analysis has a valuable role in the ED, and we used all of possible methods in our research.

### 4.1. Limitations

Our analysis possesses certain limitations that have to be acknowledged. First of all, the studied group was relatively small after the final inclusion in the study of only patients with blood gas tests performed at the ED, with data of the oxygen support at the time of blood collection. Blood gas analysis was not performed routinely in all patients but at the discretion of the treating physician. Among patients who did not receive the BG test, the overall mortality was 8.2%, potentially suggesting that there was a tendency for higher use of blood gas analysis in patients in worse conditions and a higher risk of worse outcomes. Nonetheless, in some patients, the concomitant symptoms and the results of the parallel diagnostic tests could have yielded sufficient information not to perform blood gas analysis already in the ED, but usually later on the destination ward. Furthermore, the research was based entirely on the retrospective data, from the electronic databases, which often did not define the exact hour of blood gas sampling. Some data concerning the patients' breathing support could also be inaccurate, since in case of acute dyspneic deterioration, the type of ventilation used at the time of blood sample collection may not reflect the real situation at the bedside. It can be also disputed that there may be some bias related to the hospital ward profile.

Based on these assumptions, it would be worth performing a similar prospective multicenter study which would assess the predictive value of lactate concentration on ED patient prognosis. The prospective study should also assess other end points such as hospital length of stay and ICU admission risk.

## 5. Conclusions

In our real-world analysis of patients at high risk of respiratory failure, who were admitted to the emergency department due to dyspnea, both arterial and venous blood gas lactate levels were predictive of short-term mortality. An addition of blood gas analysis to routine triage risk stratification may therefore reduce the risk of underdiagnosing emergency cardiopulmonary states. Another prospective multicenter study based on arterial and venous lactate level measurements would be beneficial for assessing the patients' prognosis but also other end points in ED patients.

## Figures and Tables

**Table 1:** Baseline patients' characteristics.

	Death	Survival	*p* value
	*n* = 38	*n* = 70	
Gender (women/men)	20/18 (52.6%/47.4%)	33/37 (47.1%/52.9%)	0.5834

Median age, years, median (Q1–Q3)	79.5 (69–87)	71 (53–80)	**0.0043**

Reason of admission, *n* (%)	Dyspnea 7 (18%)	Dyspnea 36 (51%)	**0.0009**
	Heart failure 14 (37%)	Heart failure 7 (10%)	**0.0017**
	Pneumonia 9 (24%)	Pneumonia 7 (10%)	0.0865
	ND 8 (21%)	ND 20 (29%)	0.4927

Triage level, *n* (%)	Red 13 (34%)	Red 5 (7%)	**0**.**0007**
	Orange 9 (24%)	Orange 12 (17%)	0.4511
	Yellow 15 (39%)	Yellow 49 (70%)	0.6548
	Green 1 (3%)	Green 4 (6%)	0.0038

Comorbidities, *n* (%)	Post-SCA 8 (21%)	Post-SCA 2 (3%)	**0.0034**
	DM2 17 (45%)	DM2 13 (19%)	**0.0064**
	Asthma 6 (16%)	Asthma 2 (3%)	**0.0216**
	NPL 6 (16%)	NPL 2 (3%)	**0.0216**
	CKD 13 (34%)	CKD 10 (14%)	**0.0254**
	SCA at ED 7 (18%)	SCA at ED 3 (4%)	**0.0314**
	HF 20 (53%)	HF 23 (33%),	0.0636
	COVID-19 5 (13%)	COVID-19 2 (3%)	0.0938
	OHS 1 (3%)	OHS 0 (0%)	0.3519
	ILDs 1 (3%)	ILDs 6 (9%)	0.4175
	COPD 3 (8%)	COPD 9 (13%)	0.5343
	HT 22 (58%)	HT 41 (59%),	>0.9999
	Post-ACS 6 (16%)	Post-ACS 10 (14%)	>0.9999
	Stroke 2 (5%)	Stroke 5 (7%)	>0.9999
	Post-COVID-19 1 (3%)	Post-COVID-19 1 (1%)	>0.9999
	OSA 1 (3%)	OSA 2 (3%)	>0.9999
	PE 1 (3%)	PE 1 (1%)	>0.9999

SBP median (Q1–Q3), mmHg	109 (85–130)	140 (123–167)	**<0.0001**

DBP median (Q1–Q3), mmHg	66 (46–78)	84 (70–95)	**<0.0001**

MAP median (Q1–Q3), mmHg	83 (58–93)	103 (89–118)	**<0.0001**

SpO_2_ median (Q1–Q3), %	92 (82–96)	96 (92–98)	**0.0037**

Oxygen therapy at the ED, *n* (%)	No data 3 (8%)	No data 5 (7%)	
	Own breath 9 (24%)	Own breath 36 (51%)	**0.006**
	Oxygen mask 15 (39%)	Oxygen mask 23 (33%)	0.5202
	HFNOT 2 (5%)	HFNOT 0 (0%)	0.1202
	IMV 8 (21%)	IMV 5 (7%)	0.0575
	NIV 1 (3%)	NIV 1 (1%)	>0.9999

Time spent at the ED, median (Q1–Q3), hours	6.75 (4–12)	5 (3.5–8.5)	0.21

ACS, acute coronary syndrome; CKD, chronic kidney disease; COPD, chronic obstructive pulmonary disease; DM2, diabetes mellitus type 2; DBP, diastolic blood pressure; ED, emergency department; HF, heart failure; HFNOT, high-flow nasal oxygen therapy; HT, hypertension; ILDs, interstitial lung diseases; IMV, invasive mechanical ventilation; IQR, interquartile range; NIV, noninvasive ventilation; NPL, neoplasma; ND, neurological disorders; NS, nonsignificant; OHS, obesity hypoventilation syndrome; OSA, obstructive sleep apnea; PE, pulmonary embolism; SCA, sudden cardiac arrest; SBP, systolic blood pressure. Continuous data were presented as median and IQR. Categorical data were presented as total number and %. For the clarity, in each table the *P* < 0.05 has been highlighted in bold letters.

**Table 2:** Summary of significant results from arterial blood gas test.

Arterial blood gas					
Parameter	Died	Survived	Mann–Whitney test (*p*)	Logistic regression	
				OR (95% CI)	*p*
HCO_3_^−^ median (Q1–Q3), mmol/L	20.1 (15–24.1)	25.3 (23.5–26.4)	**0.009**	0.74 (0.579–0.937)	**0.013**
BE median (Q1–Q3), mmol/L	−5.9 (−13.8- (−1.2))	1.25 (−0.9-2.3)	**0.012**	0.8 (0.661–0.959)	**0.017**
LAC median (Q1–Q3), mmol/L	3.3 (1.8–9)	1.15 (0.85–1.7)	**<0.001**	1.58 (1.013–2.470)	**0.044**

For the clarity, in each table the *P* < 0.05 has been highlighted in bold letters.

**Table 3:** ROC analysis of LAC, HCO_3_^−^, and BE from the arterial blood gas tests.

ROC ABG				
Parameter	Cutoff	Sensitivity	Specificity	AUC
LAC (mmol/L)	1.5	0.94 (0.73–0.99)	0.64 (0.41–0.83)	0.81
HCO_3_^−^ (mmol/L)	24.7	0.75 (0.51–0.91)	0.83 (0.59–0.96)	0.78
BE (mmol/L)	−0.9	0.79 (0.54–0.94)	0.78 (0.52–0.96)	0.77

**Table 4:** Summary of significant results from venous blood gas tests.

Venous blood gas					
Parameter	Died	Survived	Mann–Whitney test	Logistic regression	
				OR (95% CI)	*p*
HCO_3_^−^ median (Q1–Q3), mmol/L	21 (17.3–25.2)	24.6 (23.7–25.8)	**0.029**	0.95 (0.841–1.064)	0.356
LAC median (Q1–Q3), mmol/L	2.6 (1.5–4.7)	1.7 (1.1–2.25)	**0.014**	1.8 (1.129–2.875)	**0.013**

For the clarity, in each table the *P* < 0.05 has been highlighted in bold letters.

**Table 5:** ROC analysis of LAC, HCO_3_^−^, and BE from the venous blood gas tests.

ROC VBG	
Parameter	AUC
LAC (mmol/L)	0.68
HCO_3_^−^ (mmol/L)	0.67
BE (mmol/L)	0.65

**Table 6:** ROC analysis of LAC, HCO_3_^−^, and BE from the arterial blood gas and venous blood gas tests.

ROC ABG/VBG	
Parameter	AUC
LAC (mmol/L)	0.74
HCO_3_^−^ (mmol/L)	0.71
BE (mmol/L)	0.70

**Table 7:** Analysis of the arterial blood gas, venous blood gas, and mixed ABG/VBG regression model.

		Died (15)	Survived (20)	Mann–Whitney *p*	Univariate logistic regression OR (95% CI)	*p*
ABG (known FiO_2_)	P/F median (Q1–Q3)	199.33 (88.57–277.14)	251.19 (188.86–323.1)	0.1306	0.99 (0.984–1.001)	0.094
	pO_2_ median (Q1–Q3), mmHg	62 (58.2–73.4)	63.75 (51.2–74)	0.6745	1 (0.989–1.008)	0.7798
	pCO_2_ median (Q1–Q3), mmHg	36.4 (32.1–63.9)	39.25 (35.35–42.75)	0.915	1.01 (0.969–1.048)	0.6801
	pH median (Q1–Q3)	7.38 (7.17–7.46)	7.42 (7.38–7.46)	0.1876	0.01 (0.000–1.485)	0.07
	sO_2_ median (Q1–Q3), %	92.4 (87.5–95.7)	93.85 (87.95–96.2)	0.5586	0.95 (0.869–1.041)	0.2779
	HCO_3_^−^ median (Q1–Q3), mmol/L	20.1 (15–24.1)	25.3 (23.5–26.4)	**0.0092**	0.74 (0.579–0.937)	**0.013**
	BE median (Q1–Q3), mmol/L	−5.9 (−13.8 do −1.2)	1.25 (−0.9 do 2.3)	**0.0115**	0.8 (0.661–0.959)	**0.0166**
	LAC median (Q1–Q3), mmol/L	3.3 (1.8–9)	1.15 (0.85–1.7)	**<0.0001**	1.58 (1.013–2.470)	**0.0435**

		Died (17)	Survived (45)			
VBG (known FiO_2_)	P/F median (Q1–Q3)	108.57 (87.8–127)	140 (87.62–169.52)	0.1445	0.99 (0.976–1.002)	0.1146
	pO_2_ median (Q1–Q3), mmHg	30.7 (25–38.1)	30 (23.2–36.4)	0.7635	1.02 (0.960–1.080)	0.5268
	pCO_2_ median (Q1–Q3), mmHg	44.2 (38.7–56.2)	43.7 (41.6–48.6)	0.6815	1.03 (0.958–1.111)	0.403
	pH median (Q1–Q3)	7.34 (7.26–7.39)	7.38 (7.35–7.42)	**0.0288**	1.05 (0.235–4.658)	0.9516
	sO_2_ median (Q1–Q3), %	42.6 (32.4–72.1)	56 (33.7–67.8)	0.9595	1 (0.970–1.025)	0.8482
	HCO_3_^−^ median (Q1–Q3), mmol/L	21 (17.3–25.2)	24.6 (23.7–25.8)	**0.0293**	0.95 (0.841–1.064)	0.3555
	BE median (Q1–Q3), mmol/L	−3.5 (−6.9–2.5)	1.8 (0–3.2)	**0.0465**	0.94 (0.857–1.0342)	0.2095
	LAC median (Q1–Q3), mmol/L	2.6 (1.5–4.7)	1.7 (1.1–2.25)	**0.0135**	1.8 (1.129–2.875)	**0.0134**

		Died (38)	Survived (70)			
ABG/VBG	P/F median (Q1–Q3)	126.36 (88.19–192.05)	163.81 (103–200)	0.2536	1 (0.992–1.002)	0.3663
	pO_2_ median (Q1–Q3), mmHg	43.05 (26.4–61.4)	36.35 (26.9–50.9)	0.3966	1 (0.994–1.010)	0.5153
	pCO_2_ median (Q1–Q3), mmHg	45.1 (36.4–59)	42.8 (38.5–48.4)	0.4699	1.01 (0.977–1.047)	0.5106
	pH median (Q1–Q3)	7.35 (7.17–7.41)	7.39 (7.35–7.43)	**0.0117**	0.7 (0.200–2.421)	0.5703
	sO_2_ median (Q1–Q3), %	72.45 (34.4–91.4)	67.8 (44.1–87.8)	0.8539	1.01 (0.990–1.024)	0.4065
	HCO_3_^−^ median (Q1–Q3), mmol/L	20.7 (15.6–24.5)	24.7 (23.3–26.2)	**0.0003**	0.87 (0.788–0.964)	**0.0075**
	BE median (Q1–Q3), mmol/L	−3.55 (−10.3–1.3)	1.5 (−0.9–2.9)	**0.0004**	0.89 (0.822–0.961)	**0.031**
	LAC median (Q1–Q3), mmol/L	3.15 (1.7–8)	1.5 (1.1–2.2)	**<0.0001**	1.68 (1.221–2.3064)	**0.0014**

OR, odds ratio; CI, confidence interval; P/F, PaO_2_/FiO_2_ ratio; BE, base excess; LAC, lactate. For the clarity, in each table the *P* < 0.05 has been highlighted in bold letters.

## Data Availability

The data that support the findings of this study are available on request from the corresponding author.
